# Neuroprotection with Hemoglobin-based Oxygen Carriers in Ischemic Stroke: A Narrative Review of Preclinical Studies

**DOI:** 10.31662/jmaj.2025-0600

**Published:** 2026-08-21

**Authors:** Takeo Abumiya, Teruyuki Komatsu, Miki Fujimura

**Affiliations:** 1 Department of Neurosurgery, Hokkaido University Graduate School of Medicine, Sapporo, Japan; 2 Department of Neurosurgery, Miyanomori memorial hospital, Sapporo, Japan; 3 Department of Applied Chemistry, Faculty of Science and Engineering, Chuo University, Tokyo Japan

**Keywords:** Hemoglobin-based oxygen carrier, Ischemic stroke, Neuroprotection, No-reflow phenomenon

## Abstract

While the establishment of endovascular thrombectomy has markedly improved outcomes in acute ischemic stroke, ineffective reperfusion remains a major challenge. Despite extensive preclinical research on neuroprotective strategies, this challenge persists, likely due to the inability to effectively address the no-reflow phenomenon, characterized by microvascular luminal narrowing that impedes red blood cell passage. Hemoglobin-based oxygen carriers (HBOCs), with particle sizes significantly smaller than red blood cells, offer a unique advantage over other neuroprotective agents: they can deliver oxygen to brain tissue and exert neuroprotective effects even under no-reflow conditions. This review provides a comprehensive overview of preclinical studies investigating the therapeutic application of various HBOCs in ischemic stroke and discusses future research directions in this promising field. Because most studies on the neuroprotective effects of HBOCs in cerebral ischemia have employed rodent middle cerebral artery occlusion models, this narrative review primarily focuses on studies using comparable rodent ischemic models to evaluate differences in efficacy. Although many HBOCs have demonstrated neuroprotective potential in experimental models, their clinical translatability remains limited, particularly regarding optimal dosage and administration timing. Among the HBOCs investigated, polynitroxylated PEGylated hemoglobin and hemoglobin nanoparticles have shown particularly promising neuroprotective effects when administered intravenously at low doses at or after reperfusion onset. These specific HBOCs possess antioxidant properties that inhibit methemoglobin formation, thereby preserving oxygen transport capacity. Furthermore, these antioxidant properties may directly confer neuroprotective effects. Additionally, by binding carbon monoxide (CO) instead of oxygen, they may elicit multiple protective effects similar to those observed with low-dose CO exposure. By simultaneously addressing the no-reflow phenomenon through oxygen delivery and activating multiple neuroprotective mechanisms, HBOCs hold significant promise as a multi-targeted therapeutic strategy for ischemic stroke.

## Introduction

The establishment of therapeutic evidence for thrombectomy has fundamentally changed the management of ischemic stroke over the past few decades ^(1), (2)^. While the therapeutic advance significantly improves outcomes for many patients with ischemic stroke, ineffective reperfusion remains a major challenge in clinical practice. Key factors contributing to ineffective reperfusion include initial tissue damage, reperfusion injury, and the no-reflow phenomenon ^(3), (4)^. With the increasing prevalence of recanalization events following the broad application of thrombectomy, advancing our understanding of the pathophysiology underlying the ineffective reperfusion and developing targeted neuroprotective strategies have become crucial. Despite extensive preclinical research demonstrating the neuroprotective potential of antioxidant, anti-inflammatory, and antiapoptotic agents against ineffective reperfusion ^(5), (6)^, translation of these neuroprotective agents into clinical practice has been limited. A major limitation of these agents may be attributed to their inability to effectively address the no-reflow phenomenon, a critical ischemic condition that occurs after recanalization and is increasingly recognized in clinical settings ^(7), (8), (9)^. This phenomenon is characterized by microcirculatory impairment primarily caused by microvascular luminal narrowing due to pericyte contraction, endothelial cell swelling, astrocyte end-feet edema, neutrophil adhesion to the endothelium, and intramural thrombotic debris, all of which impede red blood cell passage ^(10), (11)^. Given these conditions, hemoglobin-based oxygen carriers (HBOCs), with particle sizes smaller than red blood cells, are thought to be capable of delivering oxygen to brain tissue and exerting neuroprotective effects even during the no-reflow phenomenon (Figure 1). This unique advantage over other neuroprotective agents highlights HBOCs as promising candidates for mitigating the no-reflow phenomenon.

**Figure 1.**
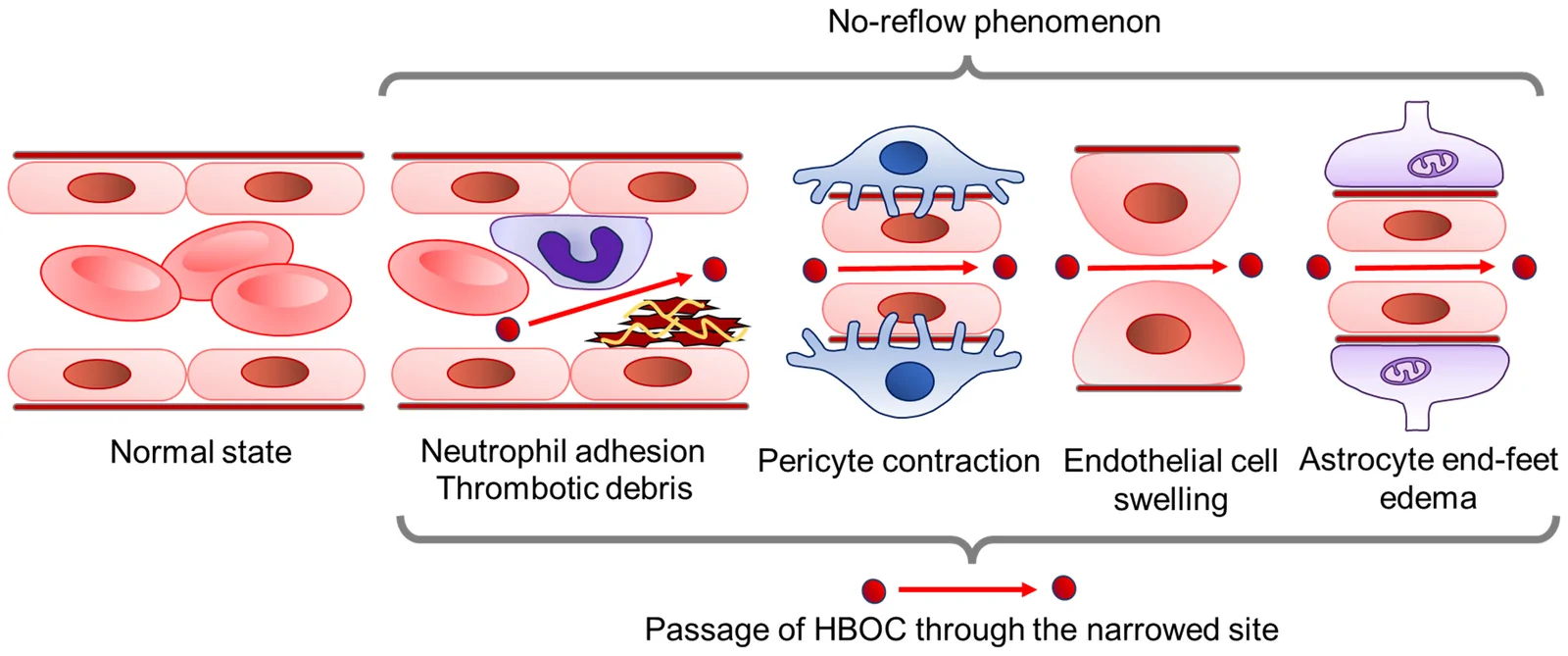
Primary effect of HBOCs in the no-reflow phenomenon. The no-reflow phenomenon is caused by microvascular luminal narrowing due to pericyte contraction, endothelial cell swelling, astrocyte end-feet edema, neutrophil adhesion to the endothelium, and intramural thrombotic debris. HBOCs, due to their smaller particle size compared to red blood cells, can pass through these narrowed sites, thereby improving microvascular perfusion and oxygen delivery. HBOC: hemoglobin-based oxygen carrier.

HBOCs have been developed as blood substitutes to address the limitations of traditional transfusions. By using hemoglobin extracted from red blood cells, HBOCs mitigate the risks of infectious diseases and blood type incompatibility. Additionally, HBOCs offer the advantage of extended storage compared to traditional blood products ^(12), (13)^. However, unmodified hemoglobin has significant drawbacks when administered directly due to its inherent toxicity ^(14)^. Notably, free hemoglobin readily binds to nitric oxide, a potent vasodilator produced by vascular endothelial cells, resulting in vasoconstriction and subsequent hypertension. Furthermore, free hemoglobin in circulation rapidly binds to haptoglobin for clearance, leading to a short half-life. Once haptoglobin stores are depleted, excess free hemoglobin accumulates in the renal tubules, potentially inducing kidney injury. To overcome these inherent limitations, various chemical modifications have been employed to enhance hemoglobin’s stability and functionality, giving rise to distinct classes of HBOCs ^(12), (13), (14)^. HBOCs can be broadly categorized into two primary types: acellular and cellular. Acellular HBOCs encompass cross-linked hemoglobin, polymerized hemoglobin, PEGylated hemoglobin, and albumin-conjugated hemoglobin, whereas cellular HBOCs primarily comprise liposome-encapsulated hemoglobin (LEH) (Figure 2). Several of these HBOCs have been used in animal transient ischemic models to assess their neuroprotective potential. This review provides a comprehensive overview of preclinical studies investigating the therapeutic application of various types of HBOCs in ischemic stroke and discusses future research directions in this promising field.

**Figure 2.**
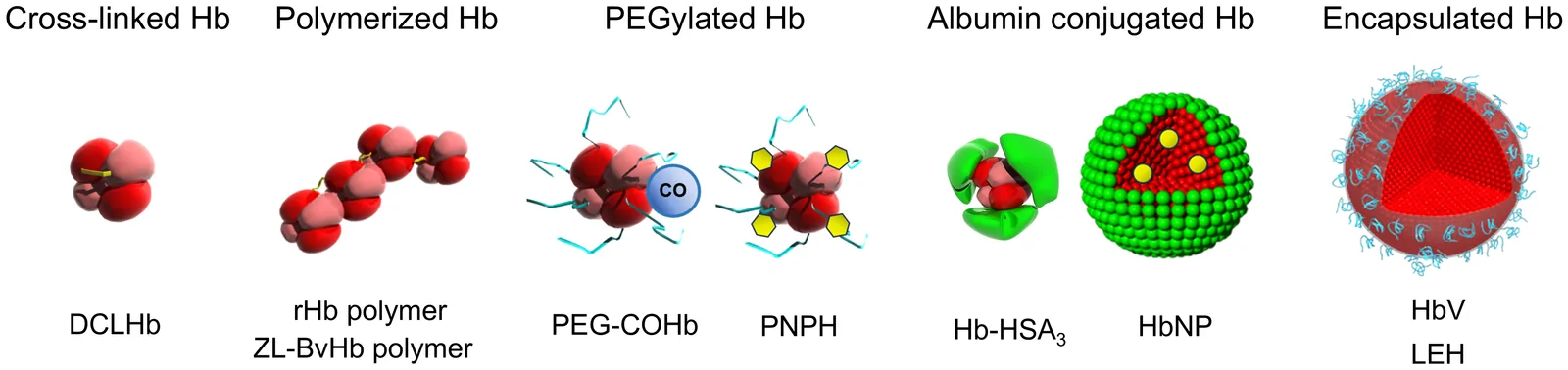
Illustrations of the structures of each HBOC. CO: carbon monoxide; DCLHb: diaspirin cross-linked hemoglobin; Hb: hemoglobin; Hb-HSA_3_: hemoglobin-albumin cluster; HbNP: hemoglobin nanoparticle; HBOC: hemoglobin-based oxygen carrier; HbV: hemoglobin vesicle; LEH: liposome-encapsulated hemoglobin; PEG-COHb: PEGylated carboxy hemoglobin; PNPH: polynitroxylated PEGylated hemoglobin; rHb: recombinant hemoglobin; ZL-BvHb: zero-link bovine hemoglobin.

Although this study was not a systematic review, literature identification and selection were performed in general accordance with the Preferred Reporting Items for Systematic Reviews and Meta-Analyses (PRISMA) recommendations using PubMed and Web of Science, up to July 2025. The search for papers investigating HBOCs in cerebral ischemia was performed using the following search terms: (“hemoglobin-based oxygen carrier*” OR “cross-linked hemoglobin*” OR “hemoglobin polymer*” OR “PEGylated hemoglobin*” OR “carboxyhemoglobin*” OR “encapsulated hemoglobin*”) AND (“stroke” OR “cerebral ischemi*” OR “brain infarct*” OR “middle cerebral artery occlusion”). The initial search yielded 329 records: 129 from PubMed and 200 from Web of Science. After removing duplicate records, 245 records were screened based on their titles and abstracts, and selected based on the inclusion criteria of being preclinical animal administration studies written in English. Additional relevant studies were identified by reviewing the reference lists of retrieved articles and prior reviews. Because most studies employed rodent experimental models, this narrative review primarily focused on studies using comparable rodent ischemic models to evaluate differences in the neuroprotective efficacy of various HBOCs. Ultimately, 13 studies were included to compare and discuss the effectiveness of HBOCs.

## HBOCs Investigated for the Treatment of Ischemic Stroke

### Cross-linked hemoglobin 

Diaspirin cross-linked hemoglobin (DCLHb) was investigated in a rat transient middle cerebral artery occlusion (MCAO) model with the therapeutic concept of hemodilution, which was achieved through blood removal and DCLHb transfusion, resulting in significant reduction in hematocrit levels. DCLHb-induced hemodilution reduced infarct volume in a dose-dependent manner in rats subjected to 3 hours of ischemia followed by 2 hours of reperfusion ^(15), (16)^. Further studies extended these findings, demonstrating the efficacy of DCLHb hemodilution in rats subjected to 2 hours of ischemia followed by 72 hours of reperfusion ^(17)^. However, despite promising preclinical results, a subsequent clinical trial in patients with acute ischemic stroke revealed that serious adverse events occurred in DCLHb-treated patients. DCLHb caused a rapid increase in mean arterial blood pressure, as well as severe organ edema and insufficiency ^(18)^. As a result, therapeutic research on DCLHb for ischemic stroke was ultimately discontinued.

### Polymerized hemoglobin

Two types of polymerized hemoglobin were evaluated for their efficacy in a murine transient MCAO model. The first agent consisted of recombinant hemoglobin polymers composed of the human alpha-subunit and the bovine beta-subunit ^(19)^. The second agent was composed of zero-link bovine hemoglobin polymers stabilized by pseudopeptide bonds ^(20)^. Both polymerized hemoglobin agents were tested for their therapeutic effects in mice subjected to 2 hours of ischemia followed by 24 hours of reperfusion. When administered after blood withdrawal as part of the hemodilution therapeutic approach, both agents significantly reduced infarct volume compared to controls ^(19), (20)^. However, subsequent studies investigating the use of polymerized hemoglobin for the treatment of ischemic stroke have been limited in number.

### PEGylated hemoglobin 

PEGylation, specifically the conjugation of polyethylene glycol to proteins, enhances protein stability, reduces antigenicity and toxicity, protects against proteolytic degradation, and extends circulatory half-life. S-nitrosylated PEGylated hemoglobin, which is presumed to exhibit nitric oxide–like activity, has been evaluated for its neuroprotective effects in a rat model of photochemical thrombosis. Although S-nitrosylated hemoglobin did not result in significant improvements in neurological outcomes, it effectively reduced the size of cortical infarct ^(21)^.

PEGylated carboxy hemoglobin (PEG-COHb) has been designed as both a carbon monoxide (CO)–releasing molecule and an oxygen carrier. Initially, PEG-COHb releases CO, which, at low concentrations, exerts vasodilatory, anti-inflammatory, and anti-apoptotic effects, and after the dissociation of CO, it subsequently functions as an oxygen carrier. Administration of PEG-COHb 20 minutes after ischemia onset has been shown to reduce infarct volume and improve neurological deficits in a rat model subjected to 2-hour ischemia followed by 24-hour reperfusion ^(22)^. In a subsequent study using a rat MCAO model under similar experimental conditions, PEG-COHb significantly reduced infarct volume and promoted the dilation of pial arterioles in the ischemic border zone. Notably, PEG-COHb transfusion was more effective than non-CO PEGylated hemoglobin in reducing infarct volume, sustaining cerebral vasodilation, and improving collateral perfusion, suggesting the neuroprotective effects of low-dose CO ^(23)^. The effects of PEG-COHb on brain injury and collateral flow were further examined in a 2-hour transient MCAO model in spontaneously hypertensive rats, which are characterized by reduced collateral flow due to pial vasoconstriction in the penumbra. PEG-COHb administration increased collateral flow and improved reperfusion in the ischemic core ^(24)^. Additionally, in a canine permanent MCAO model, PEG-COHb administration increased pial collateral flow and cerebral blood flow in the MCAO territory, resulting in a reduction in diffusion-weighted magnetic resonance imaging ischemic lesion at 2 hours after occlusion ^(25)^. Based on the results of these preclinical studies, a phase I clinical trial assessing the safety of PEG-COHb administration in patients with acute stroke undergoing mechanical thrombectomy was conducted. No significant safety concerns were identified, although a transient increase in systolic blood pressure (20–40 mmHg) was observed ^(26)^.

Another type of PEGylated hemoglobin, polynitroxylated PEGylated hemoglobin (PNPH), has been investigated for its neuroprotective effects in a rat MCAO model. Polynitroxylation of hemoglobin induces superoxide dismutase/catalase mimetic activity. Transfusion of PNPH at 20 minutes after MCAO maintained pial arteriolar dilation at 2 hours of MCAO and reduced infarct volume by more than 70%. A significant reduction in infarct volume was also observed when PNPH was transfused 2 hours after reperfusion onset ^(27)^. This experimental result demonstrated that PNPH was effective even when administered at the latest time point among the HBOCs studied in MCAO models, suggesting that PNPH has the widest therapeutic time window.

### Albumin-conjugated hemoglobin 

An albumin-conjugated hemoglobin was created by covalently biding albumin around hemoglobin, resulting in a core-shell structure. The first type of albumin-conjugated hemoglobin consisted of a single hemoglobin core bound with three human serum albumins (hemoglobin-albumin cluster [Hb-HSA_3_]). The neuroprotective effects of Hb-HSA_3_ were evaluated in a rat 2-hour transient MCAO model, with Hb-HSA_3_ administered intra-arterially just prior to reperfusion. Treatment with Hb-HSA_3_ significantly improved neurological outcomes, reduced infarct volume, and mitigated inflammatory and oxidative responses. Furthermore, Hb-HSA_3_ enhanced blood flow and tissue partial oxygen pressure in the cortical penumbra during the early reperfusion phase ^(28)^. Considering the neuroprotective effect of regional cold perfusion ^(29)^, the combined therapeutic potential of regional cold perfusion and Hb-HSA_3_ were further investigated. In this study, Hb-HSA_3_, cooled to 10°C, was administered intra-arterially via the internal carotid artery immediately prior to reperfusion in the same rat MCAO model. Cold Hb-HSA_3_ perfusion therapy demonstrated additive neuroprotective effects, as indicated by improved neurological outcomes and a significant reduction in infarct volume. Furthermore, this therapy mitigated neurological deficits and reduced hemorrhagic transformation in rats subjected to 5 hours of ischemia followed by 24 hours of reperfusion, indicating a potential extension of the therapeutic time window with this approach ^(30)^.

The second type of albumin conjugated hemoglobin is a polymerized hemoglobin particle coated with a single-layer shell composed of human serum albumin, with a diameter of 90 nm, termed a hemoglobin nanoparticle (HbNP). HbNPs incorporate catalase, which is anticipated to provide antioxidant benefits. The neuroprotective effects of HbNPs were assessed in a rat model of 2-hour transient MCAO, with intravenous administration of HbNPs at the onset of reperfusion. HbNP treatment significantly reduced infarct volume, oxidative stress, leukocyte recruitment, and blood-brain barrier disruption 24 hours after reperfusion. Additionally, tissue oxygen partial pressure levels in the affected brain remained high in the HbNP-treated group 6 hours after reperfusion, whereas the Hb-HSA_3_–treated group showed a decline in tissue oxygen levels. The difference in oxygen partial pressure levels between HbNPs and Hb-HSA_3_, that is, the preservation of oxygen transport capacity in HbNPs, is likely attributed to the antioxidant activity of catalase contained in HbNPs, which inhibits methemoglobin formation and prevents the loss of oxygen transport capacity ^(31)^.

### Encapsulated hemoglobin

Although hemoglobin vesicle (HbV) and LEH differ in nomenclature, both are categorized as cellular-type HBOCs, characterized by hemoglobin encapsulated within liposomes with particle diameters ranging from 200 to 260 nm. The neuroprotective effects of HbV were evaluated in a rat model of 1-hour transient MCAO, with HbV administered at the onset of occlusion. This intervention resulted in significantly reduced infarct volume compared to saline controls ^(32)^. LEH has been extensively studied for its neuroprotective properties in ischemic stroke models. Administration of LEH demonstrated neuroprotective effects in a rat model of photochemically induced thrombosis ^(33)^ and in a rat permanent MCAO model ^(34)^. The therapeutic time window of LEH was investigated in a rat permanent MCAO model, revealing a significant reduction in infarct volume when administered within 2 hours after MCAO but not at 4 hours ^(35)^. Intra-arterial infusion of LEH immediately before reperfusion improved neurological function and reduced infarct volume in a rat transient MCAO model. Immunohistochemical analysis demonstrated superior microvascular perfusion with LEH compared to red blood cell in the ischemia-reperfusion area. Additionally, transmission electron microscopy revealed that LEH administration prevented microvascular narrowing, a common consequence of ischemia-reperfusion injury, and identified LEH particles within the microvessels ^(36)^. The neuroprotective effects of LEH were also validated in a primate transient MCAO model, which showed reductions in infarct size and improvements in the cerebral metabolic rate of oxygen ^(37)^.

## Comparison of Experimental Methods and Results in Preclinical HBOC Studies

As most of the preclinical studies evaluating the therapeutic effects of HBOCs have been conducted using rodent transient MCAO models, Table 1 presents a comparative analysis of these studies, highlighting the dosage, timing of administration, and their effects on infarct size reduction. This table provides valuable insights into the feasibility of various HBOCs for clinical application. Regarding dosage, a volume of approximately 10 mL/kg or less is recommended to avoid overloading the systemic circulation, minimizing the risk of hemodynamic complications. Regarding the timing of administration, early delivery during the ischemic phase is often difficult in real clinical practice. Therefore, administration at the time of reperfusion or during the post-reperfusion phase appears to be a more feasible option. Furthermore, intravenous administration demonstrates practical advantages due to its simplicity, rapid delivery, and ease of generalization in diverse clinical settings. Based on these considerations, PNPH and HbNPs have been identified as promising candidates for clinical translation.

**Table 1.** Comparison of Experimental Methods and Outcomes in Preclinical HBOC Studies Using Rodent MCAO Models.

Formulation	HBOC classification	HBOC particle size (nm)	Animal species	Ischemia period (hours)	Hb dosage (mg/kg)	Volumetric dosage (mL/kg)	Infusion timing	Reperfusion period (hours)	Functional outcome	Reduced infarct rate (%)	Remarks
DCLHb ^(15)^	Cross-linked	N/A	SHR	3	~3,500	~35	90 minutes before MCAO	2	N/A	30	Blood removal
DCLHb ^(17)^	Cross-linked	N/A	SHR	2	~3,500	~35	Just prior to MCAO	72	Positive; p<0.05	52	Blood removal
rHb polymer ^(19)^	Polymerized	(500 kDa)*	Mouse	2	~2,300	~38	10 minutes after MCAO	24	N/A	66	Blood removal
ZL-BvHb polymer ^(20)^	Polymerized	(500-14,000 kDa)*	Mouse	2	~2,300	~38	10 minutes after MCAO	24	N/A	37	Blood removal
PEG-COHb ^(23)^	PEGylated	13	Rat	2	400	10	20 minutes after MCAO	72	N/A	57
PEG-COHb ^(24)^	PEGylated	13	SHR	2	320	8	30 minutes after MCAO	24	N/A	30
PNPH ^(27)^	PEGylated	8	Rat	2	400	10	20 minutes after MCAO	24	N/A	~70
PNPH ^(27)^	PEGylated	8	Rat	2	400	10	2 hours after MCAR	72	N/A	68
Hb-HSA_3_^(28)^	Albumin conjugated	15	Rat	2	350	7	Just prior to MCAR	24	Positive; p<0.01	60	Intra-arterial
Hb-HSA_3_^(30)^	Albumin conjugated	15	Rat	2	300	6	Just prior to MCAR	24	Positive; p<0.01	72	Intra-arterial, 10°C
HbNP ^(31)^	Albumin conjugated	90	Rat	2	300	6	Just prior to MCAR	24	Positive; p<0.05	48
HbV ^(32)^	Encapsulated	260–270	Rat	1	1000	10	Just prior to MCAO	24	Positive; p<0.01	35
LEH ^(35)^	Encapsulated	200–250	Rat	2	300	5	30 minutes after MCAO	24	Negative	33
LEH ^(36)^	Encapsulated	200–250	Rat	2	1200	20	Just prior to MCAR	24	Positive; p<0.01	40	Intra-arterial

DCLHb: diaspirin cross-linked hemoglobin; Hb: hemoglobin; Hb-HSA_3_: hemoglobin-albumin cluster; HbNP: hemoglobin nanoparticle; HBOC: hemoglobin-based oxygen carrier; HbV: hemoglobin vesicle; LEH: liposome-encapsulated hemoglobin; MCAO: middle cerebral artery occlusion; MCAR: middle cerebral artery recanalization; N/A: not available; PEG-COHb: PEGylated carboxy hemoglobin; PNPH: polynitroxylated PEGylated hemoglobin; rHb: recombinant hemoglobin; SHR: spontaneously hypertensive rat; ZL-BvHb: zero-link bovine hemoglobin. *Particle size was not reported; the parenthetical value indicates molecular mass.

## Optimal Properties of HBOCs and Future Directions for Stroke Treatment Research

PNPH and HbNPs have demonstrated efficacy when administered intravenously at low dosages during the onset of reperfusion or thereafter. A shared characteristic of these HBOCs is their antioxidant capacity, which plays a crucial role in maintaining their oxygen-carrying function. Over time, hemoglobin in HBOCs undergoes auto-oxidation, resulting in the formation of methemoglobin, which cannot bind oxygen and consequently reduces oxygen delivery. The inclusion of antioxidant enzyme in HBOCs, such as catalase in HbNPs, effectively suppresses methemoglobin formation ^(38)^. Notably, HbNPs have shown superior oxygen-carrying capacity in brain tissue following ischemia reperfusion compared to Hb-HSA_3_, which lacks catalase. This difference in oxygen-carrying capacity between HbNPs and Hb-HSA_3_ can be attributed to the presence of catalase in HbNPs ^(31)^. Therefore, HBOCs with antioxidant properties are more effective in sustaining their primary oxygen transport capacity, particularly over extended durations.

Additionally, the antioxidant activity incorporated into HBOCs may have advantageous functions, not only by preserving oxygen-carrying capacity but also by exerting direct neuroprotective effects. Numerous antioxidants and antioxidant enzymes have been investigated and shown to mitigate oxidative stress in ischemia-reperfusion models, resulting in a reduction of injury in the affected brain ^(39), (40), (41)^. In the case of catalase, one of the antioxidant enzymes, its administration alone leads to rapid degradation and a short half-life. However, encapsulation of catalase prevents premature elimination, allowing broader distribution within affected brain tissue ^(42)^. Consequently, catalase encapsulated in HbNPs is expected to retain its enzymatic activity, thereby providing neuroprotective effects at ischemia-reperfusion sites.

Beyond their antioxidant properties, HBOCs are capable of exerting neuroprotective effects through the action of low-dose CO bound to hemoglobin, as demonstrated by PEG-COHb. During the initial administration, CO-bound HBOCs release CO, providing neuroprotective effects, and subsequently function as oxygen carriers by binding oxygen. While CO is toxic in high doses due to its interference with oxygen delivery, when administered in controlled, low doses, it exhibits beneficial effects, such as vasodilation, anti-inflammatory action, and anti-apoptotic properties ^(43), (44), (45), (46)^. Inhalation of low-dose CO reduced infarct volume in mouse models of ischemic stroke, at least partly through activation of the nuclear factor erythroid 2–related factor 2 (Nrf2) pathway ^(47), (48)^. Administration of CO-releasing molecule-3 mitigated ischemia-reperfusion injury in a transient MCAO model by suppressing neuroinflammation and alleviating blood-brain barrier disruption ^(49)^. As PEG-COHb demonstrated neuroprotective effects without adverse effects at a hemoglobin dose of 400 mg/kg in rats ^(23)^, HBOCs complexed with CO are expected to pose minimal risk of adverse effects when administered at doses below this threshold. Because CO binding is readily achieved in other HBOCs, it is considered a promising future approach to prepare CO-binding forms of PNPH and HbNPs and investigate whether this multitarget approach yields a stronger neuroprotective effect (Figure 3).

**Figure 3.**
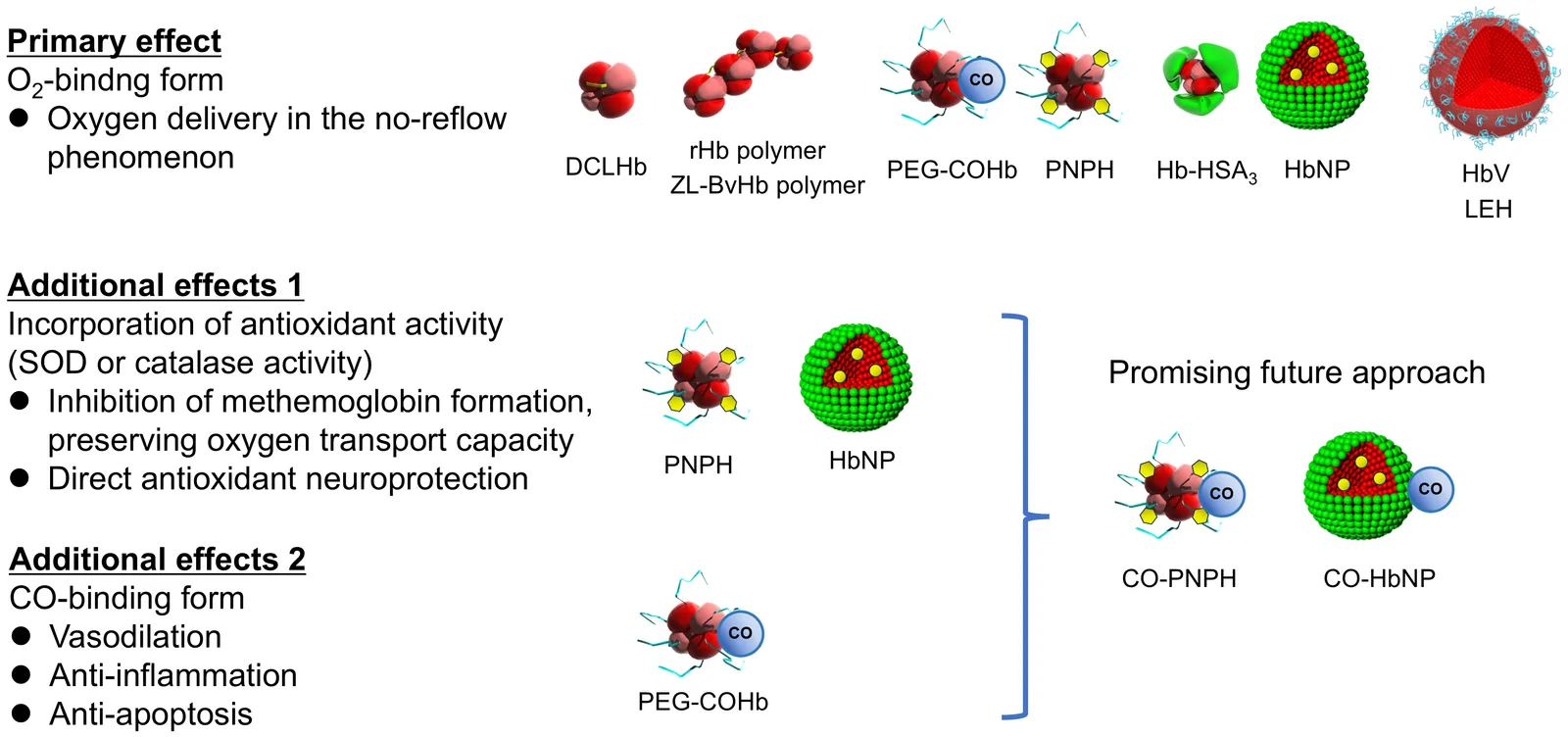
Primary and additional effects of HBOCs: multi-target therapeutic action for ischemic stroke. All HBOCs demonstrated oxygen-delivery capacity as a primary effect during the no-reflow phenomenon. PNPH and HbNPs exhibit antioxidant properties as an additional effect. PEG-COHb provides the protective effect of carbon monoxide as another additional effect. As a future approach, the investigation of a multi-target neuroprotective effect using CO-PNPH and CO-HbNP is anticipated. CO: carbon monoxide; DCLHb: diaspirin cross-linked hemoglobin; Hb: hemoglobin; Hb-HSA_3_: hemoglobin-albumin cluster; HbNP: hemoglobin nanoparticle; HBOC: hemoglobin-based oxygen carrier; HbV: hemoglobin vesicle; LEH: liposome-encapsulated hemoglobin; PEG-COHb: PEGylated carboxy hemoglobin; PNPH: polynitroxylated PEGylated hemoglobin; rHb: recombinant hemoglobin; SOD: superoxide dismutase; ZL-BvHb: zero-link bovine hemoglobin.

To enable HBOCs to function effectively under ischemia-reperfusion conditions, it is necessary to address the perfusion state of HBOCs when the blood-brain barrier is disrupted. Studies have demonstrated that microvascular disruption following ischemia reperfusion elicits the extravasation of particles approximately 10–20 nm in size, while particles larger than 30 nm are generally restricted ^(50)^. In a comparative study evaluating tissue extravasation of Hb-HSA_3_ and HbNPs at ischemia-reperfusion sites, 15 nm Hb-HSA_3_ exhibited greater extravasation compared to 90 nm HbNPs, highlighting the critical role of particle size in the retention of HBOCs within the bloodstream ^(31)^. Therefore, HBOCs with a particle size of 30 nm or lager may be more suitable for preventing HBOC leakage at disrupted microvascular lesions and mitigating the subsequent reduction in circulating HBOC volume.

To clarify the role of HBOCs in neuroprotective therapy, a comparison between HBOCs and other neuroprotective agents needs to be discussed. As mentioned in the Introduction section, although numerous neuroprotective agents have been evaluated for their neuroprotective potential in preclinical animal models, their translation into clinical practice has been limited. The vast majority of large-scale clinical trials have failed to demonstrate clinical benefit due to narrow therapeutic time windows, limitations of single-target therapies, and associated adverse effects. To date, only edaravone, a free radical scavenger, has been approved for use in Japan for acute ischemic stroke. Edaravone has long been used in clinical practice, and its safety profile is well established. However, its clinical efficacy has primarily been evaluated in studies with relatively small sample sizes, highlighting the need for validation in large, well-powered trials ^(51)^. One possible explanation for its limited efficacy is that free radical scavenging alone cannot achieve complete neuroprotection. While edaravone penetrates ischemic regions with microcirculatory disturbances, its therapeutic action is limited to antioxidant defense and fails to address the underlying hypoxia. In contrast, antioxidant-modified HBOCs offer a dual benefit by reaching these compromised regions to mitigate oxidative stress while simultaneously delivering essential oxygen. Moreover, using CO-bound antioxidant-modified HBOCs can expand these therapeutic benefits to incorporate anti-apoptotic, anti-inflammatory, and vasodilatory properties. Therefore, given their potential for multi-target therapeutic action (Figure 3), HBOCs represent a promising strategy for neuroprotective therapy in ischemic stroke.

## Limitations

Despite promising preclinical evidence, several major concerns must be addressed before advancing HBOCs to clinical translation for ischemic stroke. First, the major safety concerns associated with HBOCs include vasoconstriction caused by nitric oxide scavenging, as well as oxidative stress and renal toxicity, all of which pose significant regulatory challenges. To date, only three HBOC formulations remain under active clinical investigation in humans: Hemopure (polymerized hemoglobin), PP-007 (PEGylated hemoglobin), and NMU-HbV (encapsulated hemoglobin). Clinical trials for all other previously evaluated candidates, including HemAssist (cross-linked hemoglobin), PolyHeme (polymerized hemoglobin), Hemolink (polymerized hemoglobin), Hemospan (PEGylated hemoglobin), and Hemoximer (cross-linked hemoglobin), have been discontinued due to these adverse effects. Notably, in clinical trials involving severe trauma or cardiac surgery, some formulations demonstrated higher rates of myocardial infarction and mortality in the treatment group, significantly compromising their safety profiles. Meanwhile, although PNPH and HbNPs have shown promise in preclinical models of ischemic stroke, neither has advanced to clinical safety evaluation in humans. Furthermore, translating results from rodent ischemic models to humans remains a persistent challenge. Significant species-specific differences exist in collateral circulation profiles; white-to-gray matter ratios; variations in age, sex, and comorbidities; and the temporal dynamics of immune and inflammatory responses. Indeed, numerous neuroprotective agents that demonstrated efficacy in rodent models ultimately failed in human clinical trials. In addition, further investigation is required to establish the pharmacokinetic profiles, optimal dosing regimens, and therapeutic time windows of HBOCs. Ideally, these parameters should be evaluated in large-animal models, including non-human primates. Addressing these limitations will be essential for advancing HBOCs toward safe and effective clinical use in ischemic stroke therapy.

### Conclusions

HBOCs with small particle sizes have been extensively investigated for their potential to improve compromised microvascular perfusion in preclinical models of ischemic stroke. These studies suggest that HBOCs can exert neuroprotective effects; however, only PNPH and HbNPs have demonstrated clinical relevance in terms of both dosage and timing of administration. Both HBOCs possess antioxidant properties that not only preserve oxygen transport capacity but also directly contribute to the neuroprotection of brain tissue. Furthermore, when administered in their CO-bound forms, HBOCs may confer additional benefits, including vasodilatory, anti-inflammatory, and anti-apoptotic effects. Although several major concerns must be addressed before advancing to the clinical translation, given their potential for multi-target therapeutic action, HBOCs represent a promising strategy for neuroprotective therapy in ischemic stroke.

## Article Information

### Author Contributions

Takeo Abumiya designed the review and drafted the manuscript, Teruyuki Komatsu and Miki Fujimura revised the content, and all authors approved the final version of the manuscript.

### Conflicts of Interest

None

### Disclaimer

Miki Fujimura is one of the Editors of JMA Journal and on the journal’s Editorial Staff. He was not involved in the editorial evaluation or decision to accept this article for publication at all.

## References

[1] Goyal M, Menon BK, van Zwam WH, et al. Endovascular thrombectomy after large-vessel ischaemic stroke: a meta-analysis of individual patient data from five randomised trials. Lancet. 2016;387(10029):1723-31. 10.1016/S0140-6736(16)00163-X 26898852

[2] Xiong Y, Wakhloo AK, Fisher M. Advances in acute ischemic stroke therapy. Cir Res. 2022;130(8):1230-51. 10.1161/CIRCRESAHA.121.319948 35420919

[3] Nie X, Leng X, Miao Z, et al. Clinically ineffective reperfusion after endovascular therapy in acute ischemic stroke. Stroke. 2023;54(3):873-81. 10.1161/STROKEAHA.122.038466 36475464

[4] Elmadhoun A, Wang H, Ding Y. Impacts of futile reperfusion and reperfusion injury in acute ischemic stroke. Brain Circ. 2024;10(1):1-4. 10.4103/bc.bc_9_24 PMC11034445 38655438

[5] Dammavalam V, Lin S, Nessa S, et al. Neuroprotection during thrombectomy for acute ischemic stroke: a review of future therapies. Int J Mol Sci. 2024;25(2):891. 10.3390/ijms25020891 PMC10815099 38255965

[6] Mizuma A, You JS, Yenari MA. Targeting reperfusion injury in the age of mechanical thrombectomy. Stroke. 2018;49(7):1796-802. 10.1161/STROKEAHA.117.017286 PMC6019565 29760275

[7] Sperring CP, Savage WM, Argenziano MG, et al. No-reflow post-recanalization in acute ischemic stroke: mechanisms, measurements, and molecular markers. Stroke. 2023;54(9):2472-80. 10.1161/STROKEAHA.123.044240 37534511

[8] Mujanovic A, Ng F, Meinel TR, et al. No-reflow phenomenon in stroke patients: a systematic literature review and meta-analysis of clinical data. Int J Stroke. 2024;19(1):58-67. 10.1177/17474930231180434 37231702

[9] Horie N, Inoue M, Morimoto T, et al. Recanalization does not always equate to reperfusion: no-reflow phenomenon after successful thrombectomy. Stroke. 2025;56(1):183-9. 10.1161/STROKEAHA.124.048994 39648915

[10] Jia M, Jin F, Li S, et al. No-reflow after stroke reperfusion therapy: an emerging phenomenon to be explored. CNS Neurosci Ther. 2024;30(2):e14631. 10.1111/cns.14631 PMC10867879 38358074

[11] Sun F, Zhou J, Chen X, et al. No-reflow after recanalization in ischemic stroke: from pathomechanisms to therapeutic strategies. J Cereb Blood Flow Metab. 2024;44(6):857-80. 10.1177/0271678X241237159 PMC11318407 38420850

[12] Sen Gupta A. Hemoglobin-based oxygen carriers: current state-of-the-art and novel molecules. Shock. 2019;52(1S) Suppl 1:70-83. 10.1097/SHK.0000000000001009 PMC6874912 31513123

[13] Zhu K, Wang L, Xiao Y, et al. Nanomaterial-related hemoglobin-based oxygen carriers, with emphasis on liposome and nano-capsules, for biomedical applications: current status and future perspectives. J Nanobiotechnology. 2024;22(1):336. 10.1186/s12951-024-02606-1 PMC11180412 38880905

[14] Faggiano S, Ronda L, Bruno S, et al. From hemoglobin allostery to hemoglobin-based oxygen carriers. Mol Aspects Med. 2022;84:101050. 10.1016/j.mam.2021.101050 34776270

[15] Cole DJ, Schell RM, Drummond JC, et al. Focal cerebral ischemia in rats. Effect of hypervolemic hemodilution with diaspirin cross-linked hemoglobin versus albumin on brain injury and edema. Anesthesiology. 1993;78(2):335-42. 8439029

[16] Cole DJ, Drummond JC, Patel PM, et al. Effects of viscosity and oxygen content on cerebral blood flow in ischemic and normal rat brain. J Neurol Sci. 1994;124(1):15-20. 10.1016/0022-510x(94)90004-3 7931415

[17] Cole DJ, Drummond JC, Patel PM, et al. Hypervolemic-hemodilution during cerebral ischemia in rats: effect of diaspirin cross-linked hemoglobin (DCLHb) on neurologic outcome and infarct volume. J Neurosurg Anesthesiol. 1997;9(1):44-50. 9016440

[18] Saxena R, Wijnhoud AD, Carton H, et al. Controlled safety study of a hemoglobin-based oxygen carrier, DCLHb, in acute ischemic stroke. Stroke. 1999;30(5):993-6. 10.1161/01.str.30.5.993 10229733

[19] Nemoto M, Mito T, Brinigar WS, et al. Salvage of focal cerebral ischemic damage by transfusion of high O2-affinity recombinant hemoglobin polymers in mouse. J Appl Physiol (1985). 2006;100(5):1688-91. 10.1152/japplphysiol.01374.2005 PMC1764455 16424069

[20] Mito T, Nemoto M, Kwansa H, et al. Decreased damage from transient focal cerebral ischemia by transfusion of zero-link hemoglobin polymers in mouse. Stroke. 2009;40(1):278-84. 10.1161/STROKEAHA.108.526731 PMC2612731 18988905

[21] Kawaguchi AT, Nakai K, Fukumoto D, et al. S-nitrosylated pegylated hemoglobin reduces the size of cerebral infarction in rats. Artif Organs. 2009;33(2):183-8. 10.1111/j.1525-1594.2008.00705.x 19178465

[22] Klaus JA, Kibler KK, Abuchowski A, et al. Early treatment of transient focal cerebral ischemia with bovine pegylated carboxy hemoglobin transfusion. Artif Cells Blood Substit Immobil Biotechnol. 2010;38(5):223-9. 10.3109/10731199.2010.488635 PMC3121099 20486873

[23] Zhang J, Cao S, Kwansa H, et al. Transfusion of hemoglobin-based oxygen carriers in the carboxy state is beneficial during transient focal cerebral ischemia. J Appl Physiol (1985). 2012;113(11):1709-17. 10.1152/japplphysiol.01079.2012 PMC3544507 23042910

[24] Cipolla MJ, Linfante I, Abuchowski A, et al. Pharmacologically increasing collateral perfusion during acute stroke using a carboxyhemoglobin gas transfer agent (Sanguinate™) in spontaneously hypertensive rats. J Cereb Blood Flow Metab. 2018;38(5):755-66. 10.1177/0271678X17705567 PMC5987934 28436705

[25] Christoforidis GA, Saadat N, Liu M, et al. Effect of early Sanguinate (pegylated carboxyhemoglobin bovine) infusion on cerebral blood flow to the ischemic core in experimental middle cerebral artery occlusion. J Neurointerv Surg. 2022;14(12):1253-7. 10.1136/neurintsurg-2021-018239 PMC12379620 34907008

[26] Linfante I, Clark W, Haussen DC, et al. HEMERA‐1 carboxyhemoglobin OxygEn delivery for Evascularization in acute stroke: a prospective, randomized phase 1 clinical trial. Stroke Vasc Interv Neurol. 2024;4(4):e001246. 10.1161/SVIN.123.001246 PMC12778537 41585378

[27] Cao S, Zhang J, Ma L, et al. Transfusion of polynitroxylated pegylated hemoglobin stabilizes pial arterial dilation and decreases infarct volume after transient middle cerebral artery occlusion. J Am Heart Assoc. 2017;6(9):e006505. 10.1161/JAHA.117.006505 PMC5634295 28899897

[28] Gekka M, Abumiya T, Komatsu T, et al. Novel hemoglobin-based oxygen carrier bound with albumin shows neuroprotection with possible antioxidant effects. Stroke. 2018;49(8):1960-8. 10.1161/STROKEAHA.118.021467 29991658

[29] Kurisu K, Abumiya T, Nakamura H, et al. Transarterial regional brain hypothermia inhibits acute Aquaporin-4 surge and sequential microvascular events in ischemia/reperfusion injury. Neurosurgery. 2016;79(1):125-34. 10.1227/NEU.0000000000001088 26516820

[30] Ito Y, Abumiya T, Komatsu T, et al. Neuroprotective effects of combination therapy of regional cold perfusion and hemoglobin-based oxygen carrier administration on rat transient cerebral ischemia. Brain Res. 2020;1746:147012. 10.1016/j.brainres.2020.147012 32652148

[31] Tatezawa R, Abumiya T, Ito Y, et al. Neuroprotective effects of a hemoglobin-based oxygen carrier (stroma-free hemoglobin nanoparticle) on ischemia reperfusion injury. Brain Res. 2023;1821:148592. 10.1016/j.brainres.2023.148592 37748569

[32] Komatsu H, Furuya T, Sato N, et al. Effect of hemoglobin vesicle, a cellular-type artificial oxygen carrier, on middle cerebral artery occlusion- and arachidonic acid-induced stroke models in rats. Neurosci Lett. 2007;421(2):121-5. 10.1016/j.neulet.2007.04.080 17566655

[33] Kawaguchi AT, Fukumoto D, Haida M, et al. Liposome-encapsulated hemoglobin reduces the size of cerebral infarction in the rat: evaluation with photochemically induced thrombosis of the middle cerebral artery. Stroke. 2007;38(5):1626-32. 10.1161/STROKEAHA.106.467290 17395856

[34] Kawaguchi AT, Kurita D, Furuya H, et al. Liposome-encapsulated hemoglobin alleviates brain edema after permanent occlusion of the middle cerebral artery in rats. Artif Organs. 2009;33(2):153-8. 10.1111/j.1525-1594.2008.00700.x 19178460

[35] Kaneda S, Ishizuka T, Sekiguchi A, et al. Efficacy of liposome-encapsulated hemoglobin in a rat model of cerebral ischemia. Artif Organs. 2014;38(8):650-5. 10.1111/aor.12358 25132081

[36] Shimbo D, Abumiya T, Kurisu K, et al. Superior microvascular perfusion of infused liposome-encapsulated hemoglobin prior to reductions in infarctions after transient focal cerebral ischemia. J Stroke Cerebrovasc Dis. 2017;26(12):2994-3003. 10.1016/j.jstrokecerebrovasdis.2017.07.026 28843805

[37] Kawaguchi AT, Haida M, Yamano M, et al. Liposome-encapsulated hemoglobin ameliorates ischemic stroke in nonhuman primates: an acute study. J Pharmacol Exp Ther. 2010;332(2):429-36. 10.1124/jpet.109.160051 19910538

[38] Okamoto W, Hasegawa M, Kohyama N, et al. Core-shell structured hemoglobin nanoparticles as artificial O2 carriers. ACS Appl Bio Mater. 2022;5(12):5844-53. 10.1021/acsabm.2c00813 36399036

[39] Yun X, Maximov VD, Yu J, et al. Nanoparticles for targeted delivery of antioxidant enzymes to the brain after cerebral ischemia and reperfusion injury. J Cereb Blood Flow Metab. 2013;33(4):583-92. 10.1038/jcbfm.2012.209 PMC3618396 23385198

[40] Hou Z, Brenner JS. Developing targeted antioxidant nanomedicines for ischemic penumbra: novel strategies in treating brain ischemia-reperfusion injury. Redox Biol. 2024;73:103185. 10.1016/j.redox.2024.103185 PMC11127604 38759419

[41] Lochhead JJ, Ronaldson PT, Davis TP. The role of oxidative stress in blood-brain barrier disruption during ischemic stroke: antioxidants in clinical trials. Biochem Pharmacol. 2024;228:116186. 10.1016/j.bcp.2024.116186 PMC11410550 38561092

[42] Joseph A, Nyambura CW, Bondurant D, et al. Formulation and efficacy of catalase-loaded nanoparticles for the treatment of neonatal hypoxic-ischemic encephalopathy. Pharmaceutics. 2021;13(8):1131. 10.3390/pharmaceutics13081131 PMC8400001 34452092

[43] Otterbein LE, Bach FH, Alam J, et al. Carbon monoxide has anti-inflammatory effects involving the mitogen-activated protein kinase pathway. Nat Med. 2000;6(4):422-8. 10.1038/74680 10742149

[44] Otterbein LE. The evolution of carbon monoxide into medicine. Respir Care. 2009;54(7):925-32. 10.4187/002013209793800394 19558742

[45] Bauer I, Pannen BH. Bench-to-bedside review: carbon monoxide--from mitochondrial poisoning to therapeutic use. Crit Care. 2009;13(4):220. 10.1186/cc7887 PMC2750131 19691819

[46] Goebel U, Wollborn J. Carbon monoxide in intensive care medicine-time to start the therapeutic application?! Intensive Care Med Exp. 2020;8(1):2. 10.1186/s40635-020-0292-8 PMC6952485 31919605

[47] Zeynalov E, Doré S. Low doses of carbon monoxide protect against experimental focal brain ischemia. Neurotox Res. 2009;15(2):133-7. 10.1007/s12640-009-9014-4 PMC2719876 19384576

[48] Wang B, Cao W, Biswal S, et al. Carbon monoxide-activated Nrf2 pathway leads to protection against permanent focal cerebral ischemia. Stroke. 2011;42(9):2605-10. 10.1161/STROKEAHA.110.607101 PMC3278075 21852618

[49] Wang J, Zhang D, Fu X, et al. Carbon monoxide-releasing molecule-3 protects against ischemic stroke by suppressing neuroinflammation and alleviating blood-brain barrier disruption. J Neuroinflamm. 2018;15(1):188. 10.1186/s12974-018-1226-1 PMC6014004 29929562

[50] Cai X, Bandla A, Mao D, et al. Biocompatible red fluorescent organic nanoparticles with tunable size and aggregation-induced emission for evaluation of blood-brain barrier damage. Adv Mater. 2016;28(39):8760-5. 10.1002/adma.201601191 27511643

[51] Feng S, Yang Q, Liu M, et al. Edaravone for acute ischaemic stroke. Cochrane Database Syst Rev. 2011;2011(12):CD007230. 10.1002/14651858.CD007230.pub2 PMC12668575 22161410

